# Programmed death 1 monoclonal antibody helped to treat mixed chimeric and reactivation of Epstein-Barr virus in a patient with adult-onset chronic active Epstein-Barr virus infection after allogeneic hematopoietic stem cell transplantation: A case report

**DOI:** 10.1097/MD.0000000000028542

**Published:** 2022-01-14

**Authors:** Yahong You, Jingshi Wang, Zhao Wang

**Affiliations:** Department of Hematology, Beijing Friendship Hospital, Capital Medical University, Beijing, China.

**Keywords:** chimerism, Epstein-Barr virus, posttransplant, programmed death protein-1

## Abstract

**Rationale:**

Systemic forms of chronic active Epstein-Barr virus infection (CAEBV) can predispose a patient to a protracted course of fulminant hemophagocytic lymphohistiocytosis, which has a poor prognosis. Epstein-Barr virus (EBV) infection may persist even after theoretically curative hematopoietic stem cell transplantation.

**Patient concerns:**

A female patient with CAEBV underwent chemotherapy followed by allogeneic hematopoietic stem cell transplantation from her human leukocyte antigen-matched sister. Neutrophil and platelet engraftment was observed on day +12 and +10. Full donor chimerism (DC) was achieved on Day +21.

**Diagnoses:**

From day +38, EBV-DNA in the blood was persistently positive, and DC declined. We attempted empirical interventions such as withdrawal of immune suppression, multiple donor lymphocyte infusion, stem cell boost, and interferon-α treatment. However, EBV-DNA copies continued to increase aggressively, whereas DC decreased rapidly and then reached a nadir of 63.27%.

**Interventions:**

Salvage programmed death 1 (PD-1) antibody treatment was administered as salvage therapy at +69 and +84.

**Outcomes:**

EBV-DNA was negative on day +97 and was ultimately undetectable. Equivalently, a full and stable DC was obtained at +97.

**Lessons:**

We summarize a case of PD-1 antibody used as salvage treatment in a post-transplant patient with CAEBV, which was eradicated and full DC was obtained. This case suggests that the PD-1 antibody appears to be a promising option for fighting EBV and mixed DCs.

## 1 Introduction

Chronic active Epstein-Barr virus (CAEBV) infection is a lymphoproliferative disorder characterized by EBV^+^ T- or natural killer (NK) cell proliferation. Systemic CAEBV presents with fever, lymphadenopathy, splenomegaly, one of the life-threatening complications is fulminant hemophagocytic lymphohistiocytosis (HLH).^[1]^ Theoretically, chemotherapy followed by allogeneic hematopoietic stem cell transplantation (HSCT) is a radical cure.^[2]^ However, as the real initiator, Epstein-Barr virus (EBV) infection may remain incurable even after HSCT, with persistent EBV infection in the blood and tissue. If such a dilemma is encountered, patients will inescapably relapse.

Complete donor chimerism (DC) protects patients from late disease reactivation. A myeloablative conditioning regimen might be sufficient to cure and decrease relapse; however, 11.5% of patients achieve mixed DC.^[2]^ However, a lower level of DC is inescapably associated with recurrence, late graft failure, and poorer long-term survival.^[2–4]^ To date, many strategies have been explored to stabilize or increase donor contribution to hematopoiesis, such as withdrawal of immune suppression, multiple donor lymphocyte infusion (DLI), donor stem cell boost, interferon-α treatment, and second HSCT.^[3–5]^ However, these interventions were not satisfactory.

As an immune checkpoint blockade, programmed cell death-1 (PD-1) inhibition has achieved a good effect on patients with EBV-positive NK/T lymphoma and gastric cancer.^[6,7]^ Currently, there is evidence that the PD-1 antibody has an obvious curative effect on relapsed/refractory EBV-associated HLH. Here, we report a case of salvage treatment with PD-1 antibody, which successfully helped eliminate EBV infection and increase donor contribution.

## 2 Case presentation

**Figure 1:**
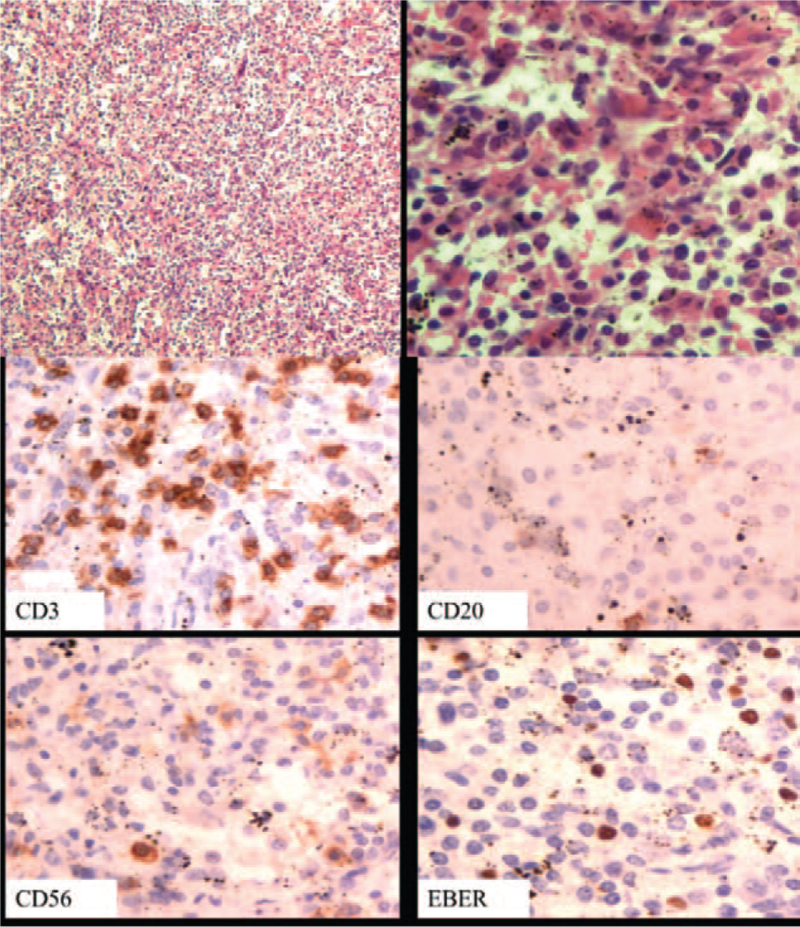
Histological study of spleen: atypical lymphocytes scattered in the background. Immunohistochemical studies and EBER-ISH of spleen: CD3+, CD20 (partial) +, CD56 (partial), and EBER-ISH+.

A 33-year-old woman was hospitalized for remittent fever and splenomegaly for >1 year. F-18 fluoro-2-deoxyglucose positron emission tomography/computed tomography showed an enlarged spleen with an abnormally increased radioactivity distribution. Laparoscopic splenectomy was then performed, and pathological examination revealed growth of atypical lymphocytes that were positive for CD3, CD20 (partial), CD56 (partial), granzyme B (partial), and TIA-1 on immunostaining. EBV-encoded small RNA (EBER) in situ hybridization was positive (Fig. 1). Histopathological examination of the spleen revealed EBV-associated T-cell lymphoproliferative disorders with EBER >50/HPF, and the Ki-67 (nuclear antigen protein) index was 10% to 20% positive. The diagnosis of CAEBV was made; EBV-DNA copies were 5.7 × 10^4^ in plasma, 1.0 × 10^7^ in peripheral blood mononuclear cells (PBMCs), and 1.4 × 10^4^ in cerebral spinal fluid (CSF). HLH was also diagnosed according to the internationally accepted guidelines of HLH-2004^[8]^: prolonged fever, splenomegaly, hemophagocytosis, hypertriglyceridemia, and decreased NK activity. No HLH-related genetic defects or other underlying conditions were observed.

We started the patient on L-DEP regimen (pegaspargase, liposomal doxorubicin with etoposide, and high-dose methylprednisolone) and then followed by DEP,^[9,10]^ and the patient achieved complete remission, intrathecal methotrexate, and dexamethasone were administrated. Fifty days after the initial diagnosis, she underwent HSCT from an HLA-identical sister.

A myeloablative conditioning regimen was employed using a total body irradiation of 900 cGy (–8, –7), a total dose of etoposide 10 mg/kg for 2 days (–6, –5), cyclophosphamide at 3.6 g/m^2^ (–4, –3), and 5.5 mg/kg rabbit anti-human thymocyte globulin (ATG, Sanofi, China) (–3, –2, –1). The number of CD34+ cells infused was 4.32 × 10^6^/kg. Neutrophil and platelet engraftment was observed on day +12 and +10. A total DC of 98.21% was achieved on Day +21.

**Figure 2:**
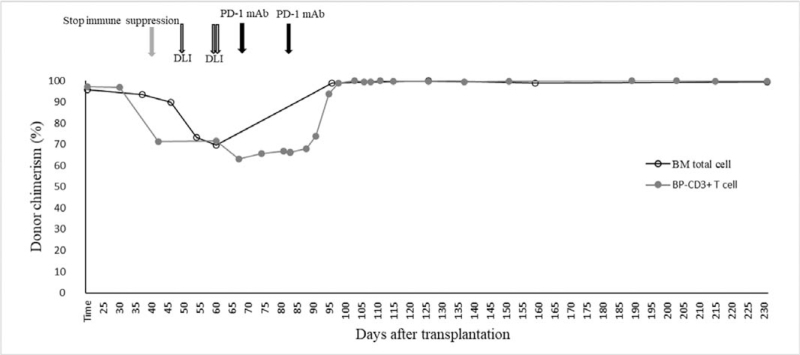
Clinical course of interventions target declined donor chimerism, lineage-specific (peripheral blood –CD3+ T cell), and total chimerism (bone marrow total cell) analysis was analyzed. BM = bone marrow, BP = blood of peripheral, DLI = donor lymphocyte infusion, PD-1 mAb = programmed death-1 monoclonal antibody.

**Figure 3:**
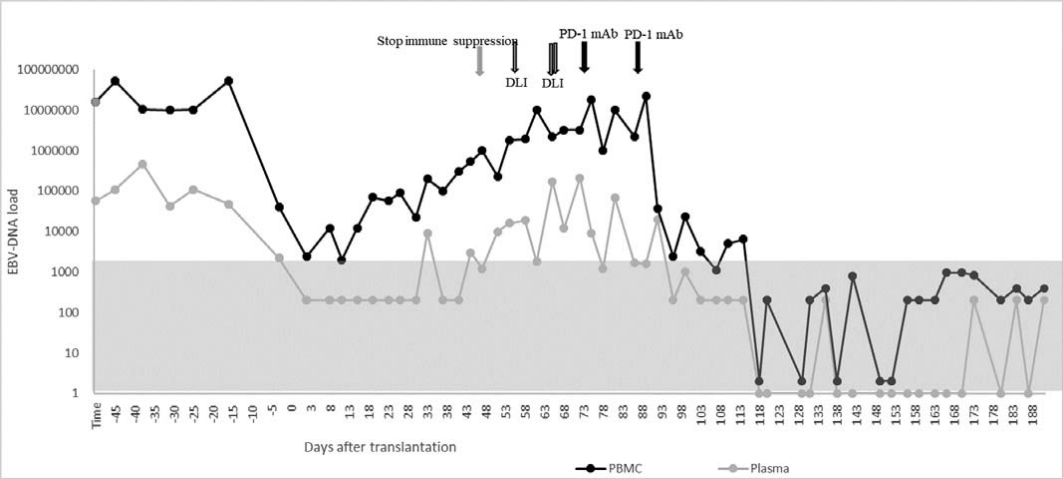
EBV-DNA load in peripheral blood, including the copies in plasma and peripheral blood mononuclear cell (PBMC). Gray zone means that the EBV-DNA was negative (below than 500 copies/mL), and dots fall on the X-axis means that EBV-DNA was radically not detectable. DLI = donor lymphocyte infusion, PD-1 mAb = programmed death-1 monoclonal antibody.

**Figure 4:**
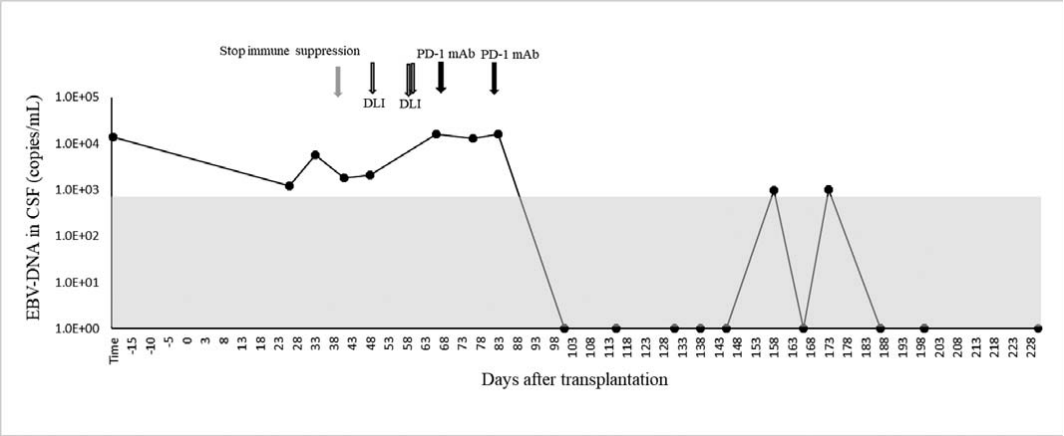
EBV-DNA load in cerebrospinal fluid during the treatment. Gray zone means that the EBV-DNA was not detectable. DLI = donor lymphocyte infusion, PD-1 mAb = programmed death-1 monoclonal antibody.

EBV-DNA copies reached their nadir in peripheral blood on day +13, after which the load began to increase. Not coincidentally, DC started to decline from day +38 and fell to 71.41% on day +42; immune suppression was stopped immediately; however, the DC continued its slide. To interfere with mixed chimerism, multiple DLIs were administered on day +47, +59, and +60. Even so, a nadir DC of 63.27% was found on day +68. The more troublesome problem was that EBV-DNA copies started to rise simultaneously. Cryopreserved donor CD34+ cells were infused on day +68 as a stem cell boost. At this time as well, a single dose of 200 mg PD-1 monoclonal antibody (Sintilimab; Suchow Xinda Biotechnology Co.; Ltd., Suchow, China) treatment was followed. Subsequently, the DC stopped falling and was maintained at approximately 65%. From day +74, recombinant human IFN-α injections (Anferon; Tianjin Hualida Biotechnology Co., Ltd., Tianjin, China) were administered at dosages of 3 million units every alternate day for 16 days. However, the DC did not move up, and EBV-DNA copies continued to rise again. The same dose of PD-1 inhibitor was applied on day +84, and acute graft versus host disease was observed on day +90; thereafter, the DC rose rapidly and converted to complete DC from day +96 (Fig. 2). It was incredible that EBV-DNA load in both plasma and PBMC were collapsed simultaneously, then it converted to negative (<5.0 × 10^2^copies/mL) and ultimately undetectable (Fig. 3). Equivalently, EBV infection in the CSF was completely eradicated (Fig. 4). The correlation of EBV-DNA load between PBMCs and plasma was evaluated, and it was positively correlated with a relative index of 0.864 (*P* < .001).

The patient developed acute graft versus host disease grade III on day +90, the main manifestations were restricted to skin rash and abnormal liver function, which were relieved after immunosuppressants. Infectious complications were effectively controlled, and no significant grade 3 or higher immune-related adverse events were observed. One year after transplantation, the patient showed no indication of relapse or acute graft versus host disease activity, and EBV-DNA was still undetectable in the plasma, PBMC, and CSF. In addition, full DC was sustained.

All procedures involving human participants were approved by the Beijing Friendship Hospital Affiliated Capital Medical University Bioethics Committee in accordance with the Declaration of Helsinki (revised in 2013). Written informed consent was obtained from the families of the patients for the publication of the case and related images.

## 3 Discussion

EBV is primarily a B-cell lymphotropic virus but has the potential to infect T and NK cells. EBV-positive gastric cancer evades T-cell immunity through PD-1/PD-L1 signal routing.^[11]^ In keeping with this dilemma, NK and CD8^+^ T cells ineffectual play a dominant part in persistent EBV infection and HLH. EBV-associated HLH often relapsed even after allo-HSCT, especially in patients with persistent EBV infection, lower donor contribution, and CNS involvement.^[3,4]^ Therefore, eliminating EBV infection is a difficult problem. In our case, EBV-DNA copies in the blood and CSF were progressively elevated, followed by a decline in DC, and in turn, the climb of EBV-DNA load was even steeper. Weaning of immune suppression, DLI, donor stem cell boost, and interferon-α treatment, all of these mixed DC-directed interventions can insufficiently avert the worst situation. A second HSCT may need to be considered even at the cost of a high risk of death and a heavier financial burden. What is startling, however, is that salvage use of PD-1 inhibitors helped to clear EBV infection and achieve a fully stable DC.

PD-1 inhibition has achieved a remarkable response in various cancers, especially EBV-positive lymphoma and gastric cancer.^[6,7,11]^ It is believed to reverse EBV-or cancer-mediated immunosuppression, thereby restoring immune function. Moreover, recent data show that PD-1 inhibition can expand PD-1 positive T cells and restore the expression of costimulatory genes in T cells.^[12]^ PD-1 inhibition may restore immunity and release T and NK cells, which would provide long-lasting benefits for patients. In our case, the load of EBV progressively increased, followed by a decrease in donor contribution, and the EBV copies collapsed immediately after the second treatment with PD-1 antibody. Even one year after HSCT, the EBV-DNA load was negative in both the peripheral blood and cerebrospinal fluid. Consistent with a recent study,^[12]^ we demonstrated that PD-1 inhibition can completely eradicate EBV even in post-transplant patients.

Mixed DCs, a persistent and increasing number of host cells after HSCT, are a predictor of hematological disease relapse.^[13–15]^ Immune modulation, via weaning immune suppression, using DLI and interferon-α, is used to change the state of mixing DCs, and can also prevent relapse in mixed DC patients.^[15,16]^ Another important thing to note is that not all patients respond well to the above therapy in a relatively short term, with the median conversion time from first DLI to full DC being 80 days (range, 28–651 days); otherwise, a second transplantation was needed.^[13]^ As an immune checkpoint inhibitor, the PD-1 inhibitor was effective in improving donor contribution. In our case, the time from the first day of immune modulation to full DCs was 44 days. Therefore, our case illustrates the potential efficacy of the PD-1 antibody in facilitating the transition from mixed DCs to full DCs.

To date, this is the first case of a PD-1 inhibitor used in a post-transplant patient with CAEBV, and remarkable results have been achieved. Traditional therapies, including transplantation, are less effective in clearing EBV. This case indicates that PD-1 inhibitor is highly effective in CAEBV: eradicate EBV in peripheral blood and CSF. Facilitate the improvement of donor contribution and convert mixed DCs to full DCs. Safe and tolerable to avoid troublesome second HSCT. Contribution to relapse-free survival.

## Acknowledgments

The authors sincerely thank the patients and their family members. The authors would also like to thank the doctors and nurses in the Department of Hematology at the Beijing Friendship Hospital, Capital Medical University. They also thank the hematologists, radiation, laboratory, pathologists, and all other participating departments for their sustained scientific collaborations.

## Author contributions

**Data curation:** Yahong You, Jingshi Wang.

**Investigation:** Yahong You.

**Methodology:** Jingshi Wang.

**Project administration:** Zhao Wang.

**Software:** Yahong You.

**Supervision:** Zhao Wang.

**Visualization:** Zhao Wang.

**Writing – original draft:** Yahong You.

**Writing – review & editing:** Jingshi Wang.
